# Stronger effects of simultaneous warming and precipitation increase than the individual factor on soil bacterial community composition and assembly processes in an alpine grassland

**DOI:** 10.3389/fmicb.2023.1237850

**Published:** 2023-08-30

**Authors:** Xiaoting Wei, Bing Han, Bo Wu, Xinqing Shao, Yongqiang Qian

**Affiliations:** ^1^Institute of Ecological Conservation and Restoration, Chinese Academy of Forestry, Beijing, China; ^2^College of Grassland Science and Technology, China Agricultural University, Beijing, China

**Keywords:** soil bacterial communities, life-history strategies, assembly processes, warming, precipitation increase, alpine grassland

## Abstract

Composition and traits of soil microbial communities that closely related to their ecological functions received extensive attention in the context of climate changes. We investigated the responses of soil bacterial community structure, traits, and functional genes to the individual warming, precipitation increases, and the combination of warming and precipitation increases in an alpine grassland in the Qinghai-Tibet Plateau that is experiencing warming and wetting climate change. Soil properties, plant diversity and biomass were measured, and the ecological processes and environmental factors driving bacterial community changes were further explored. Results indicated that the Shannon diversity of soil bacterial communities decreased significantly only under the combination treatment, which might due to the decreased plant diversity. Soil bacterial community composition was significantly correlated with soil pH, and was affected obviously by the combination treatment. At the taxonomic classification, the relative abundance of Xanthobacteraceae and Beijerinckiaceae increased 127.67 and 107.62%, while the relative abundance of Rubrobacteriaceae and Micromonosporaceae decreased 78.29 and 54.72% under the combination treatment. Functional genes related to nitrogen and phosphorus transformation were enhanced in the combination treatment. Furthermore, weighted mean ribosomal operon copy numbers that positively correlated with plant aboveground biomass increased remarkably in the combination treatment, indicating a trend of life-history strategies shift from oligotrophic to copiotrophic. Stochastic processes dominated soil bacterial community, and the proportion of stochasticity increased under the combination treatment. Our study highlights the significant effects of simultaneous warming and precipitation increase on soil bacterial community.

## Introduction

Climate warming poses a great threat to global biological diversity and ecosystem functions (Klein et al., 2004; Dawson et al., 2011). The Intergovernmental Panel on Climate Changes (IPCC, 2021) predicts that the global average temperature is expected to rise by 1.5–2°C by the end of the 21st century, and temperature increases above 2°C may lead to irreversible changes in natural ecosystems (Mann, 2009; Chen and Zhou, 2016). Climate warming has been reported to be more pronounced at higher latitudes, making these areas hotspots for studying the impacts of climate change on terrestrial ecosystems (IPCC, 2021). In addition, precipitation patterns are changing due to climate warming, and exhibit significant regional differences (Heisler and Weltzin, 2006; Park et al., 2016). The Qinghai-Tibet Plateau, known as the “the world’s third pole” is characterized by alpine ecosystem with high altitude and low temperature. It has been reported that temperature raising in this region is more rapidly than the global average level (Kang et al., 2010). Grassland is the largest ecosystem on the Qinghai-Tibet Plateau with area of 1.2 × 10^8^ hm^2^, covering 54–70% of the total area (Wang et al., 2022). Alpine grassland ecosystem is more fragile and sensitive to climate change. Vegetation productivity, plant diversity and species composition, as well as soil properties of grassland are affected obviously by warming and precipitation changes (Wang et al., 2022).

Soil microorganisms are not only important drivers of biogeochemical cycles in terrestrial ecosystems but also regulators of plant diversity and productivity (van der Heijden et al., 2008; Wagg et al., 2014; Shen and Zhao, 2015). Soil microbial communities play critical roles for ecosystem multifunctionality and resistance to climate change (Delgado-Baquerizo et al., 2017). Soil microbial community structure are closely related to edaphic factors, climate conditions as well as vegetation characteristics (Fierer, 2017; Chi et al., 2021). Variations in diversity and composition of soil microbial community based on the high-throughput sequencing in the context of global warming and precipitation pattern changing have been extensively studied in farmland, forest and grassland ecosystems. Warming and precipitation increase could affect soil microbial community composition directly or indirectly by changing soil nutrients and vegetation community (Zhang et al., 2013; Chen W. et al., 2021). The sensitivity of soil microbial communities to climate change largely depends on the vegetation composition and climatic conditions of study sites (Cregger et al., 2014; Yang et al., 2021; Jiang et al., 2022). For example, enhanced precipitation reduced soil bacterial diversity of alpine steppe not alpine meadow (Zhang et al., 2016). Climate warming had little effect on soil bacterial community composition in a Californian grassland (Gao Y. et al., 2021), while significant influences were observed in a tall-grass prairie ecosystem (Guo et al., 2018).

Recently, microbial community traits that are closely associated with ecological functions have garnered wide interest among microbial ecologists (Nakov et al., 2018; Ling et al., 2022; Yang et al., 2023). The introduction of the copiotroph-oligotroph concept to microbial ecology helps to better understand the link between the structure and function of soil bacterial communities (Kearns and Shade, 2018). Rapid growth of microbes requires more ribosome content and more rRNA gene copies in their genomes. Copiotrophs are supposed to encode more rRNA copies than oligotrophs (Roller et al., 2016), and they grow faster and are more abundant in easily decomposable carbon resources, while oligotrophs grow slower and are more adaptable to stable carbon resources with lower availability (Fierer et al., 2007). Acidobacteria, which negatively correlates with carbon mineralization rate, could be assigned to oligotrophic, while Proteobacteria and Bacteroidetes show copiotrophic properties (Fierer et al., 2007). Therefore, changes in the taxonomic composition of microbial communities might disturb the balance of life-history strategies.

The community assembly theory provides a basis for explaining changes in community diversity and composition (Dumbrell et al., 2010; Feng et al., 2018), and the stochastic-deterministic processes dichotomy is widely adopted in microbial ecology to explain the mechanisms of microbial community assembly processes (Dini-Andreote et al., 2015; Wei et al., 2022). Deterministic processes refer to biotic and abiotic environmental factors, while stochastic processes refer to some stochastic events such as dispersal, transgenation, and genetic drift (Vellend, 2010; Chase and Myers, 2011). And the relative importance of stochastic and deterministic processes is usually context dependent. For example, the relative importance of dispersal effects was greater at low latitudes than at high latitudes in maize soils across Eastern China (Jiao et al., 2020). The balance of stochastic and deterministic processes could be regulated by environmental factors such as soil pH and soil organic carbon (Dini-Andreote et al., 2015). It has been reported that warming increased the importance of deterministic processes in subtropical forest ecosystems (Zhou et al., 2023). Disentangling the driving mechanisms would facilitate our understanding of the variation of soil microbial communities under climate changes.

To enhance our comprehension of soil microbial community structures, traits, and functions under climate changes, we conducted a field experiment in alpine grasslands located in the northeast of Qinghai-Tibet Plateau, China, which simulated experiment of warming and precipitation increases was carried out for 6 years. Our study aimed to (1) investigate the impacts of individual warming, precipitation increase, and their combination on soil bacterial community composition and attributes, (2) reveal the driving mechanisms of bacterial community changes caused by climate changes, and (3) explore ecological factors that affect the diversity and composition of soil bacterial community. Climate warming alleviated the cold limitation but reduced the surface soil moisture content, exacerbating water limitation (Liu et al., 2009). Therefore, warming may interact with precipitation increases to affect soil microbial communities (Nielsen and Ball, 2015; Zhang et al., 2015). We hypothesized that (1) the combination of warming and precipitation had a synergistic effect on soil bacterial community diversity and composition, and (2) deterministic processes dominated soil bacterial community under the combination treatment due to changes of soil properties and vegetation composition.

## Materials and methods

### Study site and experimental design

The study was conducted in an alpine meadow located in the Haibei Autonomous Prefecture, Qinghai province, China (36^°^55′ N, 100^°^57′ E; 3,029 m above sea level). This region is characterized by a plateau continental climate with long cold winters and short cool summers. The mean annual temperature is 1.4°C, with monthly temperatures ranging from 17°C (June to August) to −16°C (December to January of the following year). The mean annual precipitation is 410 mm, with 80% of rainfall occurring during plant growth season (June to September). The mean annual temperature and precipitation of the study site have been increasing since 1976. The vegetation communities in the area are dominated by perennial grasses, sedges, and forbs, including *Poa crymophila* Keng, *Elymus nutans, Stipa purpurea*, *Potentilla chinensis* Ser., *Melissilus ruthenicus*, and *Kobresia humilis*. The soil is slightly alkaline with a pH range of 7.7–7.8 and is classified as a Mat-Gryic Cambisol.

In 2014, 16 round plots (diameter 2.2 m) were distributed in 4 rows and 4 columns at the study site. Four treatments were applied, including a control (CK) group, individual warming (W), individual precipitation increases (P), and the combination of warming and precipitation increase (WP), with four replicate plots for each treatment. To achieve the desired temperature increase, Open-top chambers were used, and artificial precipitation increases equivalent to 20% of the annual rainfall was artificially added to the precipitation increase plots.

### Plant and soil sampling

Vegetation communities were investigated using a 0.5 × 0.5 m quadrat in each plot during August of 2019, 2020, and 2021. Each plant species in the quadrat was recorded, and plant richness was determined by calculating the total number of plant species. The plants were then clipped at ground level, stored by species, and dried in a constant temperature oven at 75°C for 48 h before being weighed. Total aboveground biomass was calculated as the sum of the biomass of each plant species. Soil sampling was conducted using a soil auger with a diameter of 3.5 cm at a depth of 15 cm. Three locations were randomly selected for sampling in the quadrat in each plot, and the three soil subsamples were combined into one sample after homogenization. The homogenized soil samples were divided into two parts: one was stored in a refrigerator at −80°C for soil bacterial DNA extraction, and the other was air-dried for soil property measurement.

### Measurement of soil properties

The soil pH was determined using a pH probe (Orion Star A215, Thermo-Fisher Scientific, Waltham, MA, United States) with a soil-to-deionized water ratio of 1:5. Fresh soil samples were placed in pre-weighed aluminum boxes and dried for 48 h at 105°C to determine soil water content (SW). Soil total carbon (TC) and nitrogen (TN) content were measured using an elemental auto-analyzer (Vario MAXCN; Elementar, Langenselbold, Germany). The soil total phosphorus content (TP) was measured using HClO_4_-H_2_SO_4_ colorimetry, while the soil available phosphorus (AP) was measured using Mo-Sb colorimetry after extraction with 0.5 mol/L NaHCO_3_. Soil NH_4_^+^-N and NO_3_^−^-N were determined using an AA3 flow injection analyzer (Flowsys, Ecotech, Germany).

### Soil DNA extraction and 16S rRNA genes sequencing

Soil samples collected in 2019 were utilized for the analysis of bacterial communities. Genomic DNA was extracted from the soil using the OMEGA Soil DNA Kit D5625-01 (Omega Bio-Tek, Norcross, GA, USA). The extracted DNA samples were subjected to the amplification of bacterial 16S rRNA genes. The V5-V7 region targeting primer set consisting of 799F (5′-AACMGGATTAGATACCCKG-3′) and 1193R (5′-ACGTCATCCCCACCTTCC-3′) was used for amplification. The purified PCR amplicons were sequenced with the Illumina MiSeq platform in a pair-end 2 × 250 bp sequencing format.

Sequence processing was conducted using QIIME2 (2019.4)^1^ and involved further processing of raw sequences with removed primers using the DADA2 plugin. This included filtering, denoising, merging, and removal of chimera. The resulting non-singleton amplicon sequence variants (ASVs) were aligned using MEGA-X for construction of a phylogenetic tree. Taxonomy was assigned to the ASVs with the SILVA Release 132 database. For subsequent analysis, samples were rarefied to 51,133 sequences per sample.

### Data analysis

Alpha diversity (Shannon-Wiener index and Faith’s PD index) and beta diversity (Bray–Curtis dissimilarity) of bacterial communities were estimated using the diversity plugin in QIIME2 (2019.4). The sequencing reads were deemed sufficient according to the rarefaction curve (Supplementary Figure S1). Differences in bacterial community composition among treatments were visualized in Principal Coordinates Analysis (PCoA) plots based on the Bray-Curtis distance. Significance tests were conducted using permutational multivariate analysis of variance (PERMANOVA) with the “vegan” package in R (4.2.2). To assess the assembly processes of the microbial community, we calculated the β-nearest taxon index (βNTI) and Raup-Crick index using the online IEG Statistical Analysis Pipeline^2^ (Shi et al., 2018; Ning et al., 2020). When |βNTI| > 2, deterministic processes dominated the assembly of the microbial community (βNTI > 2 indicates heterogeneous selection, and βNTI < −2 indicates homogeneous selection), whereas when |βNTI| < 2, stochastic processes dominated (Stegen et al., 2012). Stochastic processes could be further subdivided into dispersal limitation, homogenizing dispersal, drift and other undominated processes based on the Raup-Crick index (RC_bray_). RC_bray_ > 0.95 indicates dispersal limitation, RC_bray_ < −0.95 indicates homogenizing dispersal, and − 0.95 < RC_bray_ < 0.95 indicates undominated processes (drift, diversification, and others). The Normalized Stochasticity ratio (NST) was calculated to further quantize the relative contribution of stochastic processes.

To assess the life-history strategies of the microbial community (copiotrophs and oligotrophs), we calculated the community weighted mean ribosomal operon copy numbers as follows. First, we downloaded the ribosomal operon (rrn) copy number information of bacteria from the rrnDB website.^3^ Then, we obtained the taxonomic classifications of bacteria using the RDP classifier tool.^4^ The ribosomal operon copies of each ASV were calculated using the “rrnDBcorrectOTU” package in R. Finally, we calculated the weighted mean ribosomal operon copy numbers (16S rrn) by considering the abundance of ASVs in each sample (Ling et al., 2022). To extrapolate the bacterial community functions, we used PICRUSt2 (Phylogenetic Investigation of Communities by Reconstruction of Unobserved States) and the KEGG database. Functional groups were further analyzed including dormancy strategies (toxin-antitoxin, sporulation, and resuscitation-promoting factors (*rpfC*)), nitrogen cycling (nitrogen fixation, urease, nitrification, and denitrification), and phosphorus transformation (organic P-mineralization, inorganic P-solubilization, P-starvation response regulation, and P-uptake and transport system) (Han et al., 2021).

Significant tests were conducted to analyze the effects of warming, precipitation increases, and their combination on vegetation and soil properties, bacterial community diversity, functional genes, as well as the weighted mean ribosomal operon copy numbers. ANOVA and Duncan multiple comparison tests were performed in SPSS (IBM SPSS Statistics 20, Chicago, United States) at a significance level of 0.05. The Mantel test with the “vegan” package in R was used to reveal correlations between environmental factors and bacterial community composition. Pearson correlation analysis was applied to explore the relationships between bacterial alpha diversity and vegetation and soil properties. Relationships between βNTI and differences in environmental factors (based on the Euclidean distance) were analyzed according to the Mantel test. Piecewise structural equation models (SEM) conducted by the “pievewiseSEM” package of R (version 4.2.2) (Shi et al., 2022) were employed to explore the direct and indirect effect routes of warming, precipitation increase, and their combination on soil bacterial community diversity and composition. Plots were generated using GraphPad Prism 8 and Adobe Illustrator (2020).

## Results

### Effects of warming and precipitation increases on plant biomass, diversity, and soil properties

**Figure 1:**
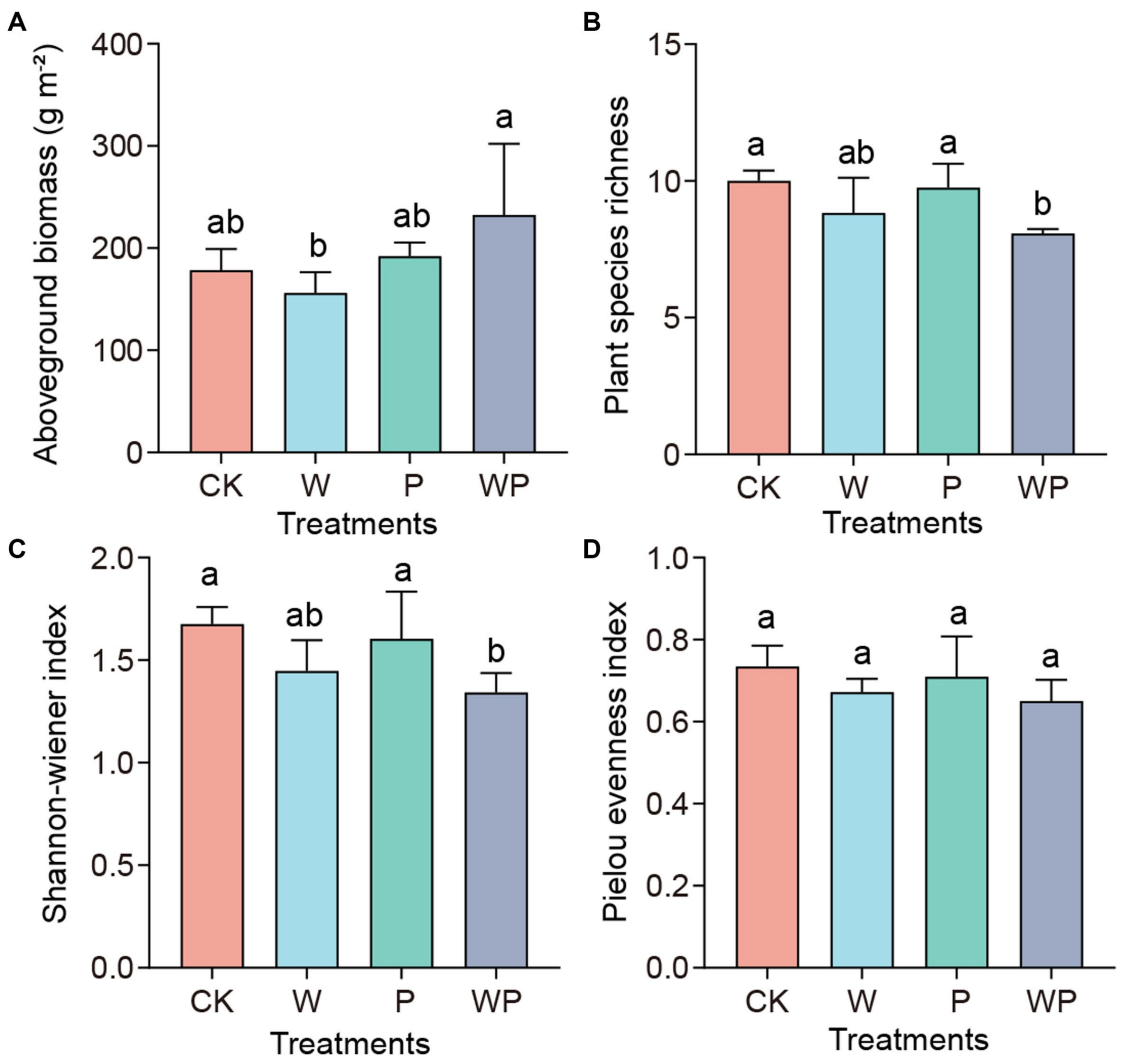
Plant aboveground biomass **(A)** and diversity **(B-D)** under different treatments. CK-control, W-warming, P-precipitation increase, WP-the combination of warming and precipitation increase. Data are shown as mean ± standard error. Different lowercase letters indicate significant differences between treatments at the 0.05 level (Duncan’s multiple comparison).

The mean vegetation aboveground biomass in the control, individual warming, precipitation increasing, as well as the combination treatment, were 178.6, 156.1, 192.2, and 232.6 g·m^−2^, respectively (Figure 1A). The significance test indicated that there were no significant differences between the control and the other three treatments (*p* > 0.05). However, the biomass under the combination treatment was significantly higher than that under the individual warming treatment (*p* < 0.05). Compared with the control treatment, there were no significant changes in plant richness and Shannon-Wiener index under individual warming and precipitation increase, but they decreased significantly under the combination treatment (Figures 1B,C). The Pielou evenness index showed bare of changes across treatments (Figure 1D).

Soil properties were also measured (Table 1). The mean soil water content in the control plots was 19.25%. It decreased in the individual warming treatment (17.05%), and increased in the individual precipitation (22.06%) and the combination treatment (20.74%). Warming and precipitation increase alone caused a slightly but significantly declined soil pH (Table 1). Soil total carbon, nitrogen, and phosphorus content showed no significant changes under the individual warming, precipitation increase, and the combination treatment (*p* > 0.05). Soil total carbon content ranged from 40.25 to 42.23 g·kg^−1^, total nitrogen content ranged from 3.38 to 3.59 g·kg^−1^, and total phosphorus content ranged from 0.22 to 0.25 g·kg^−1^ across treatments. Precipitation increases alone and the combination treatment significantly improved available nitrogen content (*p* < 0.05).

**Table 1:** Comparison of soil variables between treatments.

	CK	W	P	WP
SW/%	19.25 ± 0.18c	17.05 ± 0.31d	22.06 ± 0.34a	20.74 ± 0.48b
pH	7.83 ± 0.02a	7.70 ± 0.03b	7.72 ± 0.02b	7.78 ± 0.03ab
TC (g kg^−1^)	40.66 ± 1.15a	41.52 ± 0.34a	42.23 ± 0.59a	40.25 ± 0.78a
TN (g kg^−1^)	3.38 ± 0.07a	3.47 ± 0.07a	3.59 ± 0.07a	3.40 ± 0.06a
TP (g kg^−1^)	0.23 ± 0.01a	0.23 ± 0.02a	0.22 ± 0.02a	0.25 ± 0.00a
AP (mg kg^−1^)	2.32 ± 0.47b	2.77 ± 1.05ab	3.72 ± 0.40a	2.92 ± 0.63ab
NH_4_^+^-N (mg kg^−1^)	3.99 ± 0.29b	3.85 ± 0.16b	4.68 ± 0.82ab	5.21 ± 0.88a
NO_3_^−^-N (mg kg^−1^)	2.73 ± 0.42b	3.31 ± 0.88ab	5.73 ± 1.87a	5.80 ± 2.16a

CK-control, W-warming, P-precipitation increase, WP-the combination of warming and precipitation increase. SW-soil water content, TC-total carbon content, TN-total nitrogen content, TP-total phosphorus content, AP-available phosphorus content. Different lowercase letters indicate significant differences among treatments at the 0.05 level (Duncan’s multiple comparison).

### Effects of warming and precipitation increase on soil bacterial community diversity and composition

**Figure 2:**
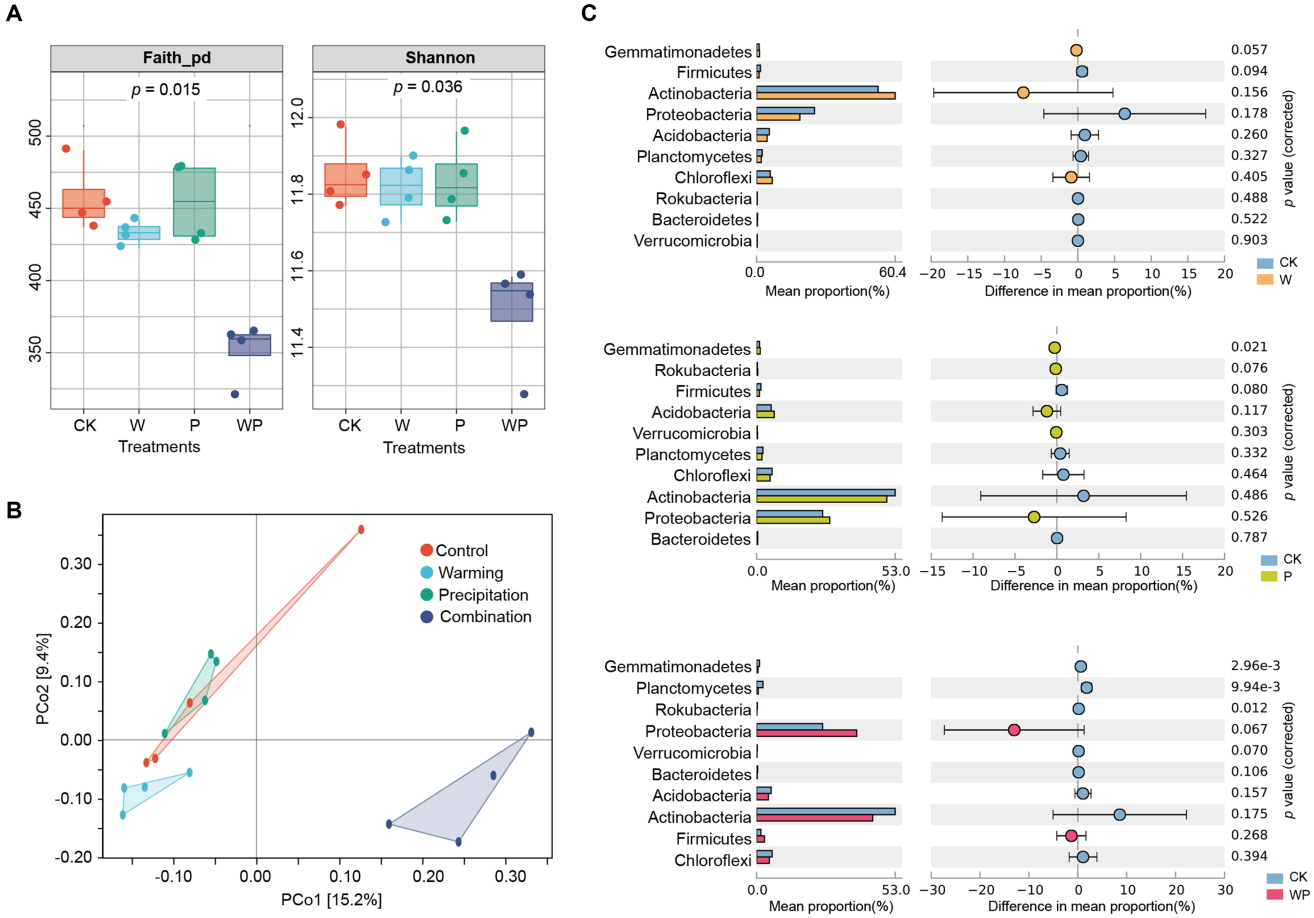
Comparison of bacterial community diversity and composition among treatments. **(A)** Alpha diversity, **(B)** Principal Co-ordinates Analysis (PCoA) plots, **(C)** relative abundance of bacterial taxa at the phylum level (graphed by STAMP, Welch’s *T* test at the 0.05 level). CK-control, W-warming, P-precipitation increase, WP-the combination of warming and precipitation increase.

Warming and precipitation increases alone had no significant effects on soil bacterial Shannon diversity and Faith_pd phylogenetic diversity (Figure 2A). However, the combination treatment caused a significant decrease in bacterial diversity (*p* < 0.05). Based on the PCoA plot, samples of the combination treatment were grouped together and were clearly separated from the other three treatments (Figure 2B). Combining this with the PERMANOVA significance test, we found that soil bacterial community structure was significantly affected by individual warming (*F* = 1.15, *p* = 0.02) and the combination treatment (*F* = 1.80, *p* = 0.03), but not precipitation increase alone (*F* = 1.16, *p* = 0.10). Additionally, there were significant differences in soil bacterial communities between warming and precipitation increase (*F* = 1.40, *p* = 0.03), warming and the combination treatment (*F* = 2.23, *p* = 0.03), as well as precipitation increase and the combination treatment (*F* = 2.05, *p* = 0.03).

For taxonomic composition, the Actinobacteria phylum was dominant, accounting for a relative abundance of 44.39–60.39% across treatments, followed by Proteobacteria with a relative abundance of 18.86–38.25% (Supplementary Figure S2). Warming alone did not significantly change the relative abundance of the main bacterial phyla and families (Figure 2C and Supplementary Figure S3). The proportion of Gemmatimonadetes increased significantly in the individual precipitation treatment. The combination treatment significantly decreased the relative abundance of Planctomycetes, Gemmatimonadetes, and Rokubacteria. At the family level, the relative abundance of Burkholderiaceae, Xanthobacteraceae, and Beijerinckiaceae increased, while the relative abundance of Rubrobacteriaceae and Micromonosporaceae decreased under the combination treatment (Supplementary Figure S3).

### Assembly processes of soil bacterial communities

**Figure 3:**
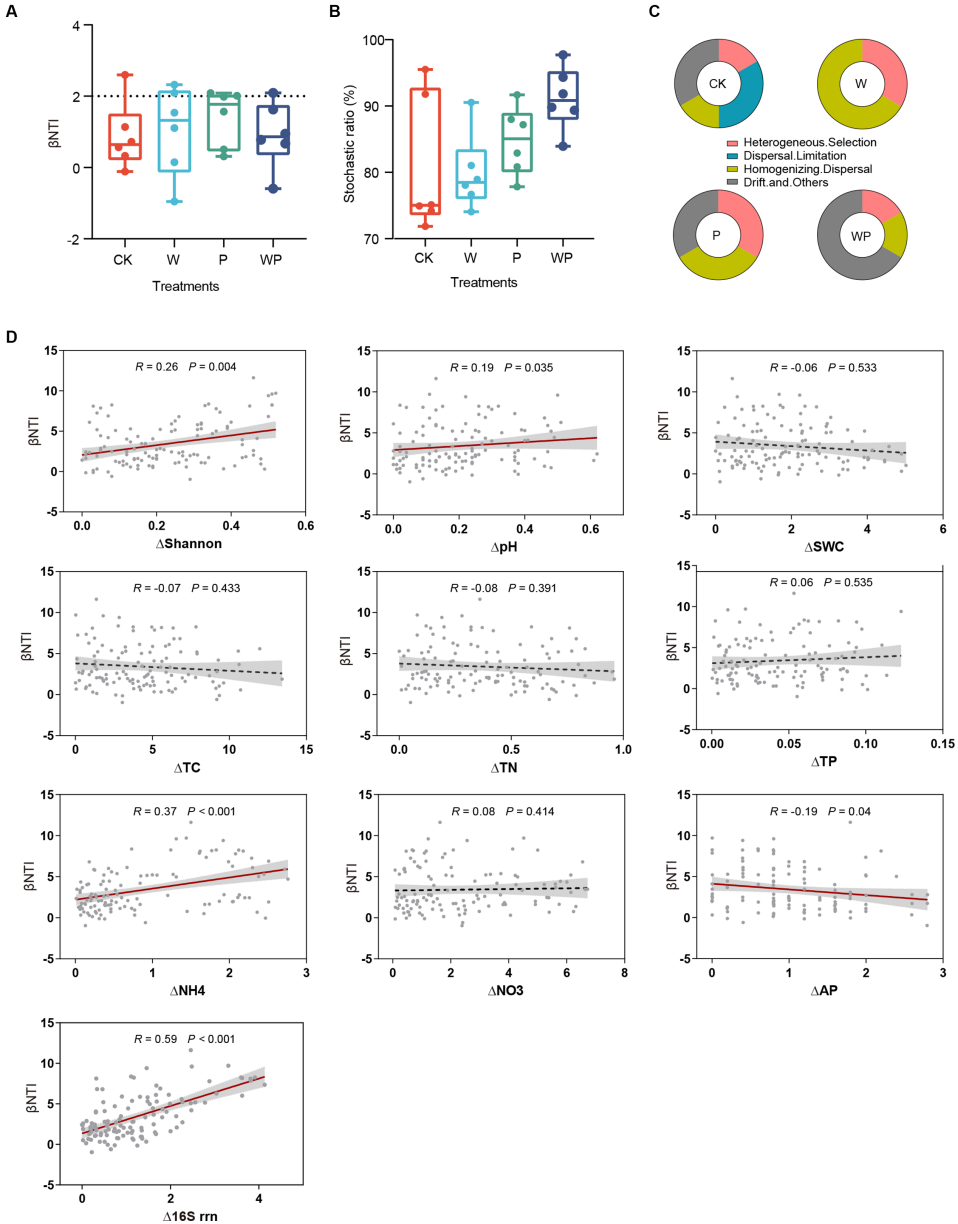
βNTI of paired samples **(A)**, the proportion of stochasticity **(B)** and ecological processes **(C)** for each treatment. Relationships between βNTI and the change degree of soil properties and life-history strategies **(D)**. CK-control, W-warming, P-precipitation increase, WP-the combination of warming and precipitation increase.

The relative contribution of deterministic and stochastic processes was evaluated based on the null model. The majority of βNTI values fell between −2 and 2, suggesting that assembly of soil bacterial communities were largely controlled by stochastic processes (Figure 3A). The proportion of stochasticity in the CK, W, P, WP was 74.18, 78.05, 82.91, and 89.82%, respectively (Figure 3B). Specifically, homogenizing dispersal dominated the bacterial communities in warming plots, homogenizing dispersal and undominated processes were equally important in precipitation increase plots, and undominated stochastic processes (drift and others) were more important in the combination plots. Dispersal limitation was observed only in the control plots (Figure 3C). The Mantel test revealing the relationship between βNTI and changes of environmental parameters was conducted. Results indicated that pairwise comparisons of βNTI were positively correlated with change degree of plant Shannon diversity index, soil pH, soil ammonium content, and negatively correlated with soil available phosphorus content (Figure 3D). In addition, a significant positive correlation between βNTI and change degree of 16S rrn was found (Figure 3D).

### Effects of warming and precipitation increase on bacterial functional genes and life-history strategies

**Figure 4:**
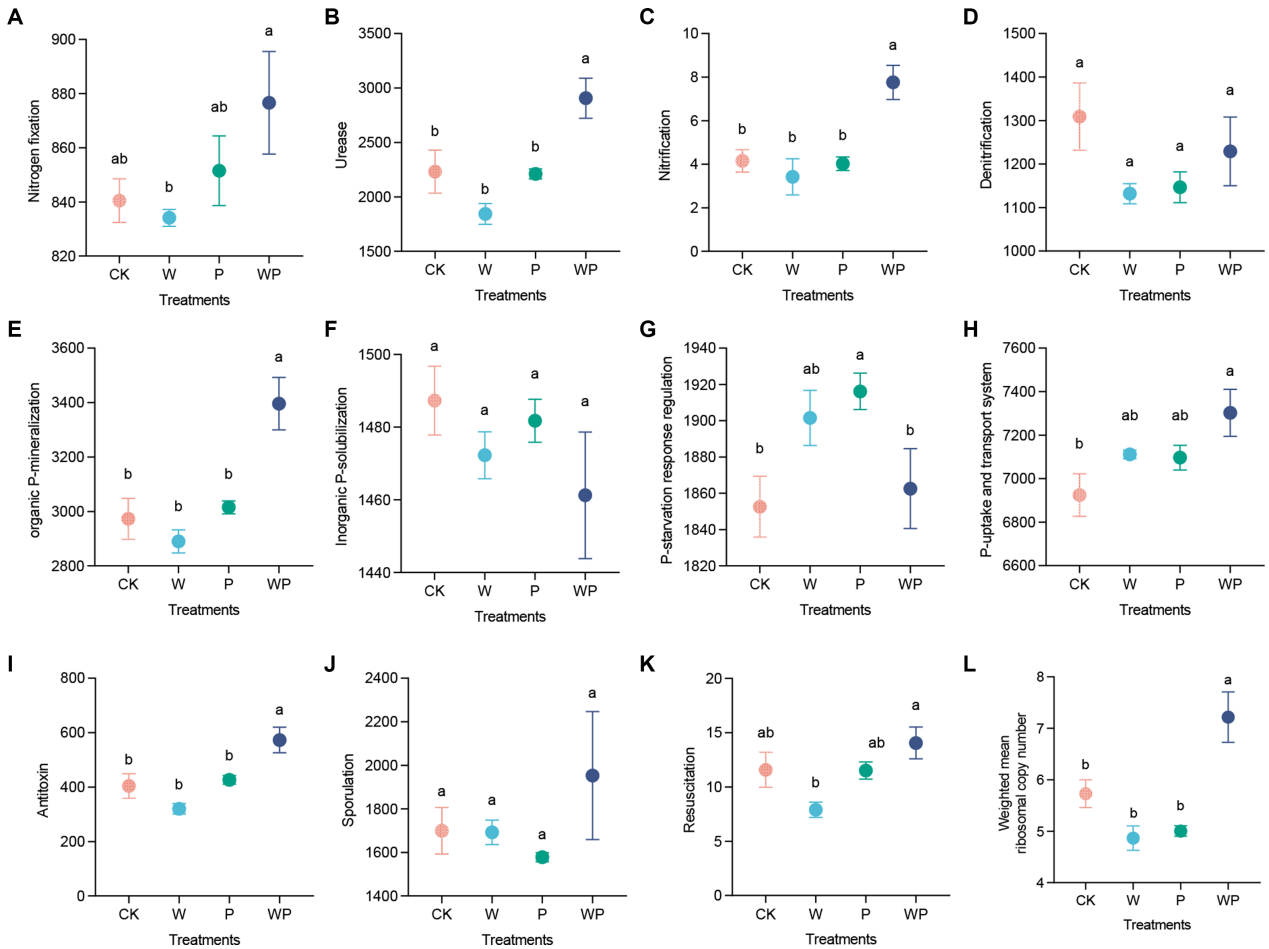
Functional genes related to nitrogen **(A–D)** and phosphorus cycling **(E–H)**, dormancy potential **(I–K)**, and weighted mean ribosomal operon copy numbers **(L)** under different treatments. Data are shown as mean ± standard error. Different lowercase letters indicate significant differences among treatments at the 0.05 level (Duncan’s multiple comparison).

The soil bacteria in the combination treatment exhibited a higher abundance of genes related to nitrogen cycling, including nitrogen fixation, ureolysis, and nitrification (Figures 4A–D). Moreover, the abundance of genes encoding P mineralization and P uptake and transport system was also enhanced by the combination treatment. Conversely, the individual precipitation treatment enhanced the abundance of genes encoding P-starvation response regulation (Figures 4E–H). Additionally, the abundance of genes encoding antitoxin under the combination treatment was significantly higher than that under the other three treatments (Figure 4I). We did not observe any significant difference in the abundance of genes encoding sporulation among treatments (Figure 4J). However, the individual warming treatment showed lower abundance of resuscitation promoting genes (Figure 4K). Furthermore, the weighted mean ribosomal operon copy numbers increased significantly under the combination treatment, while slightly decreased under the individual warming and precipitation increase treatment (Figure 4L).

### Environmental factors associated with bacterial community structure and life-history strategies

**Figure 5:**
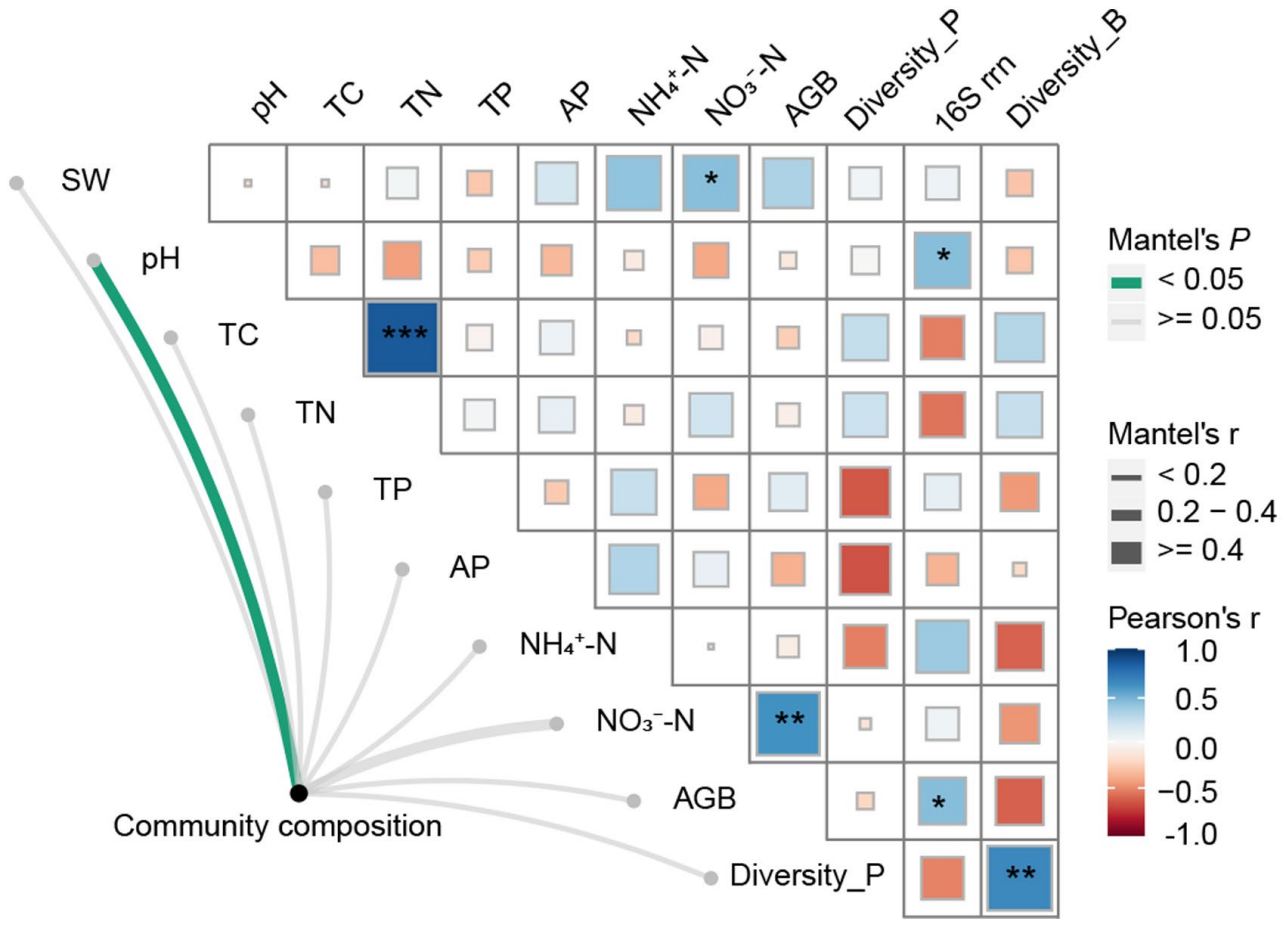
Combined heatmap showing correlations between the paired plant and soil characteristics, and their relations with bacterial community diversity, composition, and gene copy number. *Indicates significant correlation at the 0.05 level, **indicates significant correlation at the 0.01 level, and ***indicates significant correlation at the 0.001 level. SW-soil water content, pH-soil pH, TC-soil total carbon content, TN-soil total nitrogen content, TP-soil total phosphorus content, AP-soil available phosphorus content, NH_4_^+^-N-soil ammonium nitrogen content, NO_3_^−^-N-soil nitrate nitrogen content, Richness-plant richness, AGB-aboveground biomass, Diversity_P-plant diversity, Diversity_B-Bacterial diversity.

**Figure 6:**
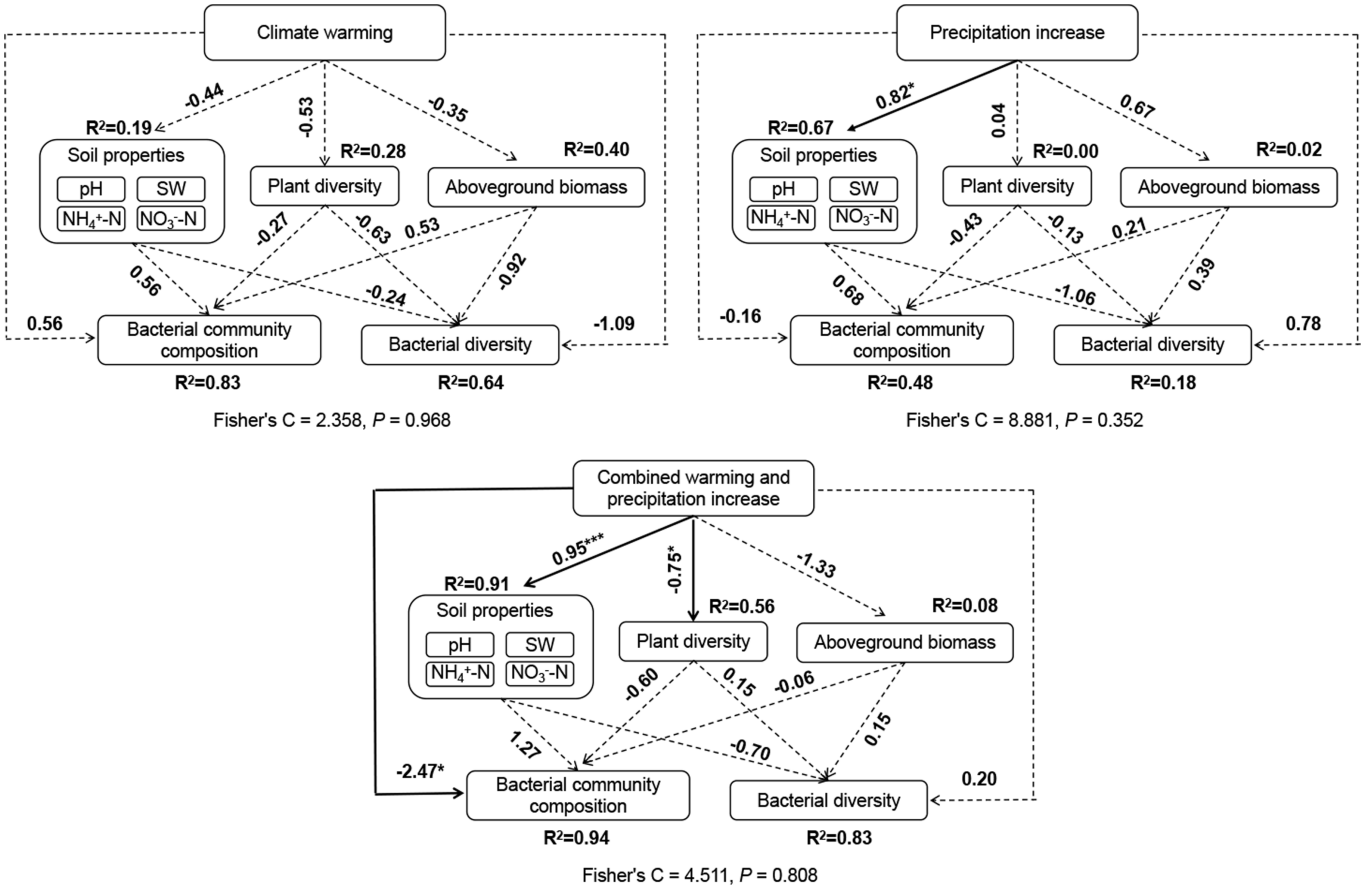
Structural equation models (SEM) revealing direct and indirect factors that affect soil bacterial community diversity and composition under the individual warming, precipitation increase, and combination of warming and precipitation increase. Solid arrows indicate significant effects (*p < 0.05, **p < 0.01, and ***p < 0.001), the dashed arrows indicate non-significant effects. Numbers on arrows represent the standard path coefficient. *R*^2^ next to the response variable represents the proportion of variation explained by dependent variables.

We observed significant positive correlations between bacterial Shannon diversity and plant diversity (Figure 5). And, bacterial community composition was significantly correlated with soil pH and AGB according to the mantel test (Figure 5). Weighted mean ribosomal operon copy numbers were positively correlated with soil pH and AGB (Figure 5). Additionally, soil NO_3_^−^-N was positively correlated with AGB and SW. SEM revealed that climate warming did not affect plant diversity and biomass, soil properties, and soil bacterial community structure directly or indirectly. Precipitation increases only affected soil properties directly. The combination of warming and precipitation increase affected plant diversity, soil properties and soil bacterial community composition by direct routes (Figure 6).

Different bacterial taxa exhibited varied responses to environmental factors (Supplementary Figure S4). Notably, the relative abundance of Xanthobacteraceae, Beijerinckiaceae, and Burkholderiaceae was positively correlated with aboveground biomass. The relative abundance of Xanthobacteraceae was also positively correlated with soil pH. Furthermore, soil nitrate nitrogen content was positively correlated with the relative abundance of Xanthobacteraceae, Sphingomonadaceae, and Rhizobiaceae, while it was negatively correlated with the relative abundance of Pseudonocardiaceae and Micromonosporaceae.

## Discussion

### The combination of warming and precipitation increase significantly changed soil bacterial community diversity and composition

Consistent with most of previous publications (Weedon et al., 2012; Zi et al., 2018; Mu et al., 2021), we found no substantial effects of individual warming on soil bacterial community alpha diversity, which further confirmed the relative stability of soil bacterial diversity in alpine meadow ecosystem under OTC warming champers. Actually, warming effects on soil microbial communities usually depend on the warming device and magnitude. A warming experiment using infrared radiator in a tallgrass prairie in the US Great Plains showed that soil bacterial diversity decreased after over 7 years +3°C warming (Wu et al., 2022). The temperature increase within the OTCs was usually smaller than 0.5°C, which might insufficiently result in changes in soil microenvironment. However, warming changed the overall bacteria community composition, even though the relative abundance of top 10 most abundant phyla remained unchanged compared with the control plots, which was in agreement with the results of another study on the Tibetan Plateau (Mu et al., 2021). Therefore, we speculated that these changes under the warming treatment might be due to some rare species. Similar to the results of warming, precipitation increase (+20%) alone had no significant effect on soil bacterial community diversity and composition. In a desert ecosystem northern China, increase in precipitation (+30 and 60%) were also unable to change soil bacterial diversity and community composition (Wu et al., 2020). The changes of soil bacterial communities are very complex and affected comprehensively by soil properties, vegetation composition and some stochastic events. Long-term continuous monitoring will be necessary to explore changes of soil bacterial communities in response to climate changes.

Different from the results of individual warming and precipitation increase, we found a significantly decrease of bacterial community diversity under the combination treatment, which indicated that the simultaneous of multiple climate change factors had stronger effects on soil bacterial communities. In addition, plant diversity was positively correlated with soil bacterial community diversity, which has been reported by previous studies (Zeng et al., 2016; Wu et al., 2020). Diverse plant species could enrich the substrate of soil microbes (van der Heijden et al., 2008). The decrease of plant diversity might be an explanation for the significant decrease of soil bacterial diversity under the combination treatment. Co-occurrence relationships among bacterial species are important factors of community diversity and composition. The increase of the relative abundance of Proteobacteria under the combination treatment might lead to the loses of some rare species.

It has been reported that the abundance of functional genes associated with nitrogen cycling changed after 14 years of warming treatment in an annual grassland ecosystem that experienced Mediterranean-climate (Gao Y. et al., 2021). This finding is inconsistent with the neutral effects of warming observed in this study, and the discrepancy may be due to differences in climate type and warming duration. Moreover, the changes in bacterial community composition and functional genes (nitrogen and phosphorus cycling) were more prominent in the combination treatment in the present study. The plant productivity and soil available nitrogen increased under the combination treatment, which could stimulate microbial activity. As substrates for nitrification, the increase in ammonium nitrogen in the interaction treatment could improve the abundance of functional genes associated with nitrification. Higher abundance of genes encoding organic phosphorus mineralization and phosphorus uptake and transport systems could be one explanation for the increase in available phosphorus (Wang et al., 2020).

Environmental factors that influence microbial community composition vary depending on the ecosystem, treatment, and sampling scale. In grasslands, soil properties are significantly correlated with soil microbial communities, while in shrublands, plants are more important in explaining changes in soil microbial communities (Chen W. et al., 2021). In crop fields, the Bray-Curtis distances of bacterial communities are strongly correlated with soil available phosphorus (Zhang et al., 2022). Soil organic matter and salinity are important factors affecting the bacterial community in riparian and coastal wetlands (Chi et al., 2021). Furthermore, many studies have emphasized the importance of soil pH in influencing bacterial community composition (Fierer and Jackson, 2006; Bartram et al., 2014), with pH being the dominant factor affecting the reconstruction of bacterial communities (Xun et al., 2015). In our study, we found a significant correlation between bacterial community composition and soil pH, with soil pH being positively correlated with the relative abundance of Xanthobacteraceae, which have been reported to have the ability to fix nitrogen (Oren, 2014). Therefore, soil pH might directly or indirectly drive the structure of bacterial communities by changing the availability of soil nutrients and affecting plant growth (Lammel et al., 2018).

### The combination of warming and precipitation increase improved copiotrophic bacteria in soil

Understanding how microbial communities respond and adapt to climate changes is critical for predicting the consequences of these changes. Soil microorganisms can adjust their life-history strategies in response to climate changes, as demonstrated in previous studies (Li et al., 2022). According to the “tolerant-sensitive-opportunistic” strategies, bacterial ecological strategies changed in response to drying and rewetting stress, and relative abundance of bacterial taxa with tolerant strategies increased while with sensitive and opportunistic strategies decreased (Evans and Wallenstein, 2014). Zi et al. (2018) reported that oligotrophic groups such as Nitrospirae increased with warming due to the reduced nutrient mobility and mineralization. In this study, we used the copiotroph-oligotroph classification and found a significant increase in gene copy number in the combination treatment, suggesting a shift toward copiotrophs. Copiotrophic bacteria with higher weighted mean ribosomal operon copy numbers grow faster than oligotrophic bacteria, leading to faster turnover of soil nutrients and improved nutrient availability (Roller et al., 2016; Chen Y. et al., 2021). We found significant positive correlations between weighted mean ribosomal operon copy numbers and aboveground biomass. Previous studies have shown that life-history strategies of soil microbial communities shift from oligotrophic to copiotrophic with vegetation restoration (Yang et al., 2023), indicating that vegetation characteristics may be important regulators of microbial life-history strategies. Microbes with different life-history strategies feed on different resource types, and trade-offs of microbial life-history strategies influence microbial residues accumulation and the turnover of soil organic matter (Shao et al., 2021).

### Stochastic processes dominated the assembly of soil bacterial community

Deterministic and stochastic processes based on the niche theory have been widely used to understand the processes underlying changes in bacterial community composition. Guo et al. (2018) found that warming significantly decreased the stochasticity of soil bacterial community assemblages in a prairie ecosystem. In a semiarid grassland, the ratio of stochasticity first increased and then decreased with increasing nitrogen application levels from 0 to 64 g·N·m^−2^ (Liu et al., 2021). Our study found that stochasticity was dominant under control, individual warming, precipitation increasing, and the combination treatment, which is consistent with the results of Ning et al. (2020). While, the proportion of stochasticity increased under the combination treatment, mainly referring to drift and other undominated processes. Studies have proved that communities with low diversity have a higher probability of gene drift (Hanson et al., 2012).

Factors regulating the balance of stochastic and deterministic processes vary across different ecosystems. In the present study, βNTI was significantly correlated with the difference in plant diversity, soil pH, soil ammonium content, as well as soil available phosphorus content. Previous studies have shown that the assembly processes of microbial communities are strongly linked to soil organic carbon and pH (Dini-Andreote et al., 2015; Fan et al., 2018; Tripathi et al., 2018). In coastal estuarine wetlands, βNTI was found to be positively correlated with soil salinity (Zhang et al., 2021), while in wetlands, the assembly processes of bacterial communities were influenced by soil water content (Gao G. F. et al., 2021). Temperature and Fe^2+^ were identified as key regulators of the balance between deterministic and stochastic processes in sediment from hot springs (He et al., 2021). Moreover, βNTI was found to be significantly associated with 16S rrn in the present study, which implied that the balance of assembly processes might affect the life-history strategies of soil bacterial community.

## Conclusion

The structure, life-history strategies, and functions of soil bacterial communities in the alpine grassland ecosystem were significantly affected by the combination of warming and precipitation increase, rather than the individual treatments. Specifically, the combination treatment decreased bacterial diversity, enhanced functional genes related to nitrogen and phosphorus transformation, and increased the weighted mean ribosomal operon copy numbers. Soil bacterial diversity was positively correlated with plant diversity, and community composition was associated with soil pH. Life-history strategies might be regulated by plant aboveground biomass. Assembly processes of soil bacterial communities were dominated by stochastic process, and the proportion of stochasticity increased under the combination treatment.

## Data availability statement

The data presented in the study are deposited in the NCBI repository, accession number PRJNA949428 (https://www.ncbi.nlm.nih.gov/bioproject/PRJNA949428).

## Author contributions

XW: investigation, writing—original draft, and visualization. BH: investigation. BW: writing—review and editing. XS: conceptualization, funding acquisition, and supervision. YQ: funding acquisition and writing—review and editing. All authors contributed to the article and approved the submitted version.

## Funding

This work was supported by the Fundamental Research Funds of Chinese Academy of Forestry (CAFYBB2022XA002 and CAFYBB2022QD001-3), and the National Natural Science Foundation of China (31971746 and 32171685).

## Conflict of interest

The authors declare that the research was conducted in the absence of any commercial or financial relationships that could be construed as a potential conflict of interest.

## Publisher’s note

All claims expressed in this article are solely those of the authors and do not necessarily represent those of their affiliated organizations, or those of the publisher, the editors and the reviewers. Any product that may be evaluated in this article, or claim that may be made by its manufacturer, is not guaranteed or endorsed by the publisher.

## Footnotes

^1^https://docs.qiime2.org/2019.4/tutorials/

^2^http://ieg3.rccc.ou.edu:8080/

^3^https://rrndb.umms.med.umich.edu/static/download/

^4^https://rrndb.umms.med.umich.edu/estimate/
