# Supplementary material for: Stronger effects of simultaneous warming and precipitation increase than the individual factor on soil bacterial community composition and assembly processes in an alpine grassland
Source: Front Microbiol. 2023 Aug 30;14:1237850. doi: 10.3389/fmicb.2023.1237850 (PMC10502225; doi:10.3389/fmicb.2023.1237850)
Supplement: Supplementary file 1 [file Data_Sheet_1.PDF]

Supplementary Material

Stronger effects of simultaneous warming and precipitation increase than the individual factor on soil bacterial community composition and assembly processes in an alpine grassland

Supplementary Figures

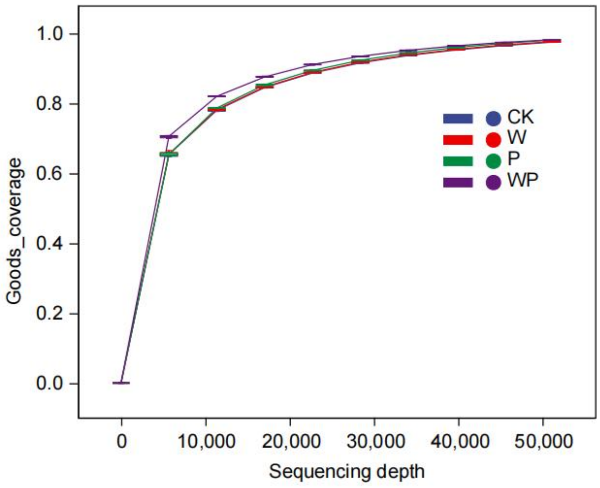

Supplementary Figure S1. Rarefaction curves of observed species under different treatments. CK-control, W-warming, P-precipitation increasing, WP-the combination of warming and precipitation increase.

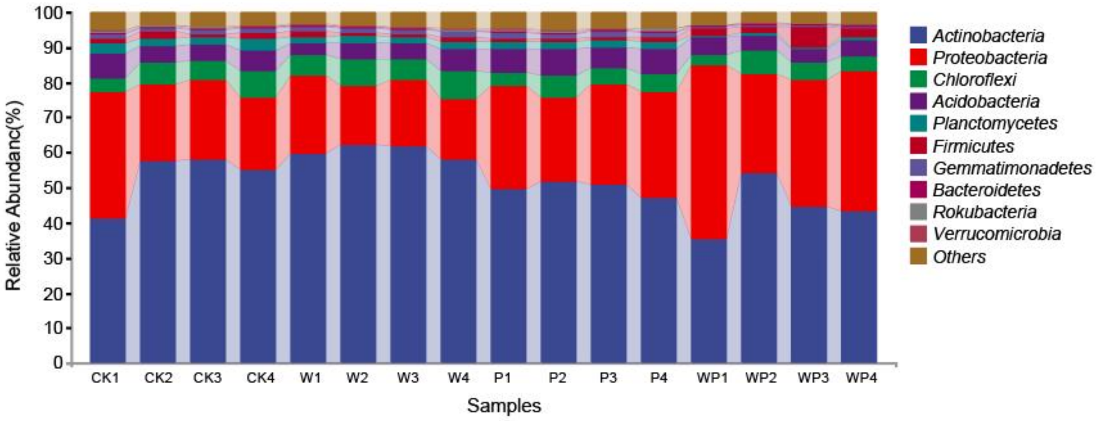

Supplementary Figure S2. Relative abundance of bacterial taxon at the phylum level. CK-control, W-warming, P-precipitation increasing, WP-the combination of warming and precipitation increase. The number represents the repetition.

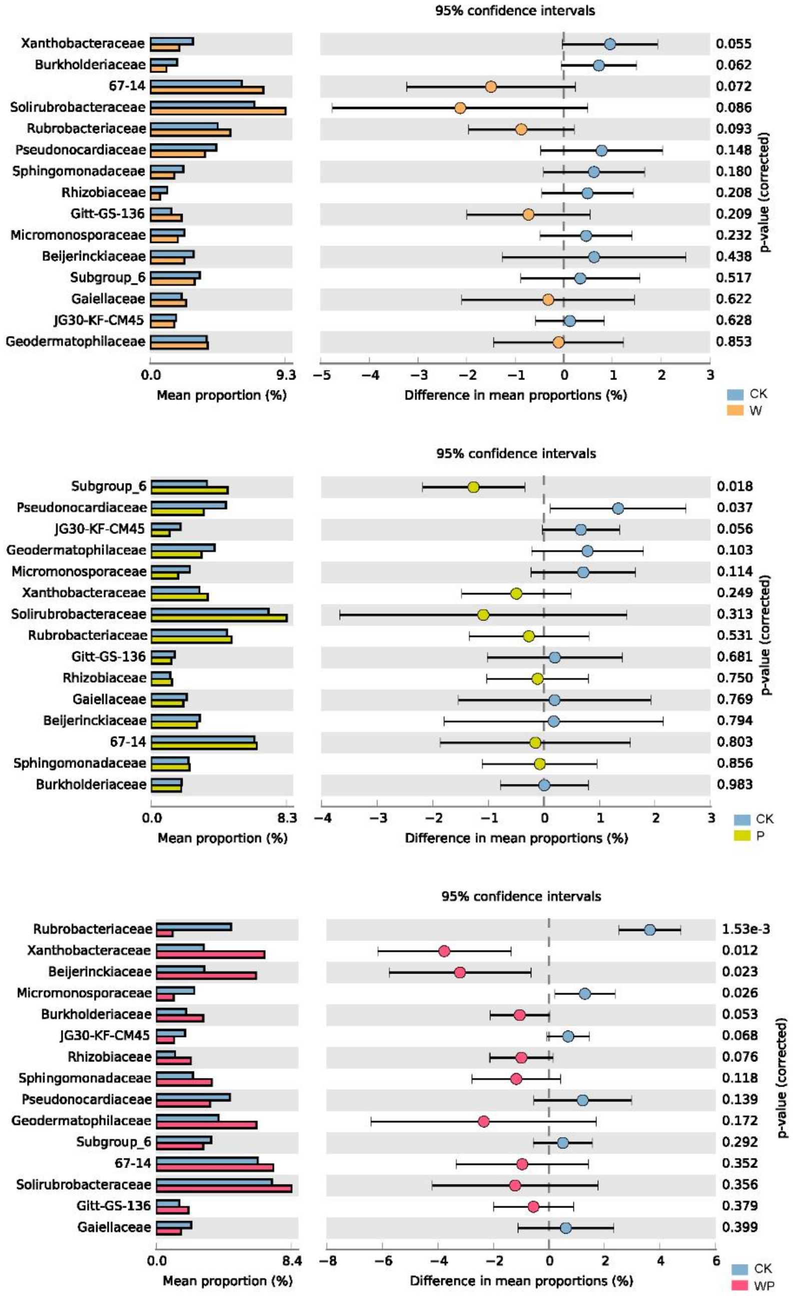

Supplementary Figure S3. Comparison of relative abundance of bacterial taxon at the family level between the control and the other three treatments. CK-control, W-warming, P-precipitation increasing, WP-the combination of warming and precipitation increase. The significance test was conducted based on the Welsh's T test at the 0.05 level.

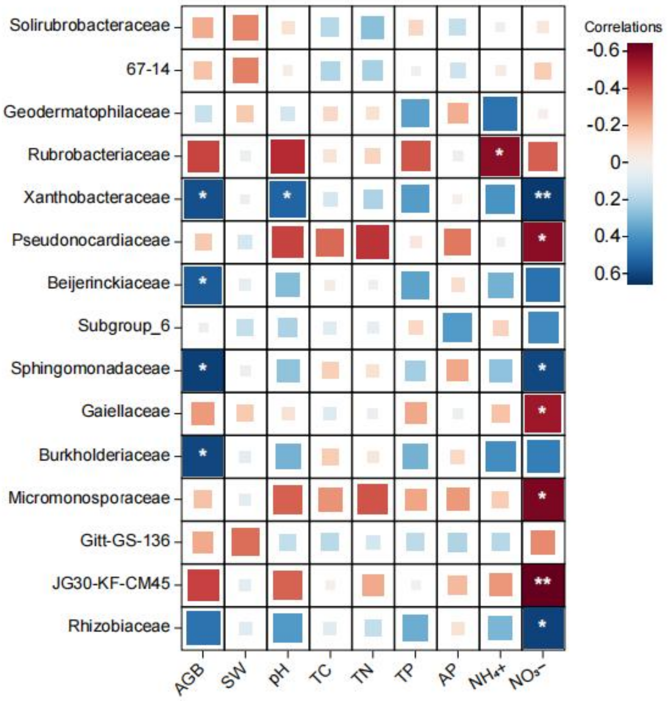

Supplementary Figure S4. Spearman correlations between the relative abundance of the top 15 bacterial families and environmental factors. AGB-Aboveground biomass, SW-soil water content, pH-soil pH, TC-soil total carbon content, TN-soil total nitrogen content, TP-soil total phosphorus content, AP- soil available phosphorus content, NH_4^+- soil ammonium nitrogen content, NO_3^-- soil nitrate nitrogen content.
